# Stroke Mechanisms in Intracranial Atherosclerotic Disease: A Modified Classification System and Clinical Implications

**DOI:** 10.1007/s12975-025-01338-0

**Published:** 2025-03-06

**Authors:** Shuang Li, Xuan Tian, Xueyan Feng, Bonaventure Ip, Hing Lung Ip, Jill Abrigo, Lina Zheng, Yuying Liu, Yu Liu, Ziqi Li, Tingjun Liang, Karen K. Y. Ma, Florence S. Y. Fan, Sze Ho Ma, Hui Fang, Bo Song, Yuming Xu, Howan Leung, Yannie O. Y. Soo, Vincent C. T. Mok, Ka Sing Wong, Xinyi Leng, Thomas W. H. Leung

**Affiliations:** 1 Department of Medicine & Therapeutics, The Chinese University of Hong Kong, Hong Kong SAR, Shatin China; 2 Department of Neurology, Beijing Tiantan Hospital, Capital Medical University, Beijing, China; 3 Department of Imaging and Interventional Radiology, The Chinese University of Hong Kong, Hong Kong SAR, China; 4 Department of Neurology, The First Affiliated Hospital of Zhengzhou University, Zhengzhou, China

**Keywords:** Intracranial atherosclerotic stenosis, Ischemic stroke, Stroke mechanisms, Stroke recurrence, Diffusion-weighted imaging

## Abstract

**Background:**

In patients with symptomatic intracranial atherosclerotic stenosis (sICAS), recent evidence has suggested an association between artery-to-artery embolism (AAE) and cortical borderzone (CBZ) infarcts.

**Methods:**

We recruited patients with 50–99% anterior-circulation sICAS in this cohort. Stroke mechanisms were categorized as isolated parent artery atherosclerosis occluding penetrating artery (PAO), isolated AAE, isolated hypoperfusion, and mixed mechanisms, using two classification systems. In Classification I, the probable stroke mechanisms of internal borderzone and CBZ infarcts were both hypoperfusion, which were respectively hypoperfusion and AAE in Classification II. Other classification criteria were the same. We investigated and compared the predictive values of the two systems in predicting 90-day and 1-year recurrent ischemic stroke in the same territory (SIT).

**Results:**

Among 145 patients (median age 62 years), 101 (69.7%) were males. We found significant difference in the proportions of baseline stroke mechanisms between these two systems (p < 0.001). Eleven (7.6%) and 19 (13.1%) patients respectively had 90-day or 1-year recurrent SIT. Classification II better predicted the risk of 90-day recurrent SIT than Classification I, when patients were divided into 4 groups according to baseline stroke mechanisms (p = 0.029), or by the presence of hypoperfusion (p < 0.001). The two classification systems had comparable predictive values for 1-year recurrent SIT.

**Conclusions:**

In medically treated sICAS patients, considering AAE rather than hypoperfusion as the stroke mechanism for CBZ infarcts could better predict early recurrent SITs.

**Supplementary Information:**

The online version contains supplementary material available at 10.1007/s12975-025-01338-0.

## Introduction

In patients with symptomatic intracranial atherosclerotic stenosis (sICAS) receiving medical treatment by current guidelines, the stroke mechanism is an important factor governing the risk of stroke recurrence [1, 2]. Probable stroke mechanisms in sICAS in the anterior circulation could be classified based on acute infarct topology in diffusion-weighted MR image (DWI), as isolated parent artery atherosclerosis occluding penetrating artery (PAO), artery-to-artery embolism (AAE), hypoperfusion, and mixed mechanisms. Under guideline-recommended medical treatment, the residual risks of stroke recurrence differed in patients with different baseline stroke mechanisms. Mixed mechanisms of AAE and hypoperfusion have been associated with a higher risk of same-territory stroke recurrence than other stroke mechanisms (24.4% versus 7.8%) [2], and presence of hypoperfusion as a stroke mechanism has also been associated with early stroke recurrence in medically treated sICAS patients [1, 3, 4].

In previous stroke mechanism classification systems for anterior-circulation large artery atherosclerotic strokes, internal (IBZ) and cortical borderzone (CBZ) infarcts are usually classified as “hypoperfusion” [2, 5]. However, there might be differences in the pathogenesis between the two infarct patterns. In a recent study, we used CT Angiography (CTA)-based computational fluid dynamics (CFD) modeling to simulate cerebral flood flow and assess the hemodynamic significance of symptomatic, atherosclerotic M1 middle cerebral artery (MCA-M1) stenosis. We found that patients with IBZ infarcts in DWI were more likely to have a larger pressure drop across the MCA-M1 lesion in the CFD model, which reflected impaired antegrade blood flow. However, presence of CBZ infarcts was not associated with the translesional pressure drop, which was associated with coexisting small cortical infarcts assessed that are probably related with AAE [4]. Moreover, under current medical treatment, we found that patients with anterior-circulation sICAS with IBZ and CBZ infarcts might have different trajectories in stroke recurrence: those with IBZ infarcts had a higher stroke risk than those with CBZ infarcts within 90 days after the index stroke, but the stroke risks later in 1 year were similar between the two groups [4].

More reasonable or accurate classification of the stroke mechanisms could help identify “high-risk” sICAS patients, which may inform secondary stroke prevention. Therefore, in this study, we modified a previous stroke mechanism classification system in anterior-circulation sICAS [2], by classifying CBZ infarcts as “AAE” rather than “hypoperfusion”. We compared the predictive values of the two classification systems for early and late stroke recurrence in medically treated sICAS patients.

## Methods

### Study Design and Subjects

This was a substudy of a prospective, longitudinal cohort study, the Stroke Risk and Hemodynamics in Intracranial Atherosclerotic Disease (SOpHIA) study, which enrolled medically treated sICAS patients [6]. The study was approved by Joint Chinese University of Hong Kong–New Territories East Cluster Clinical Research Ethics Committee (reference number: 2014.329). The study obeyed the Declaration of Helsinki, as amended by the World Medical Association General Assembly in October 2013. Informed consent was obtained from all participants. The study was reported according to the STROBE (Strengthening the Reporting of Observational Studies in Epidemiology) statement.

sICAS patients enrolled in SOpHIA study from Prince Wales Hospital in Hong Kong and the First Affiliated Hospital of Zhengzhou University in Zhengzhou were screened and analyzed in the current study with the following criteria: 1) acute ischemic stroke, attributed to 50–99% atherosclerosis stenosis of the intracranial segment of internal carotid artery (ICA) or MCA-M1; 2) acute infarct(s) shown in DWI within 14 days of stroke onset; and 3) the patient received medical treatment by latest guidelines and was followed up for 1 year. Patients were excluded with the evidence of 1) non-atherosclerotic intracranial stenosis, e.g., dissection, vasculitis; 2) cardioembolism, e.g., atrial fibrillation; 3) coexisting > 50% stenosis in ipsilateral common or internal carotid artery; 4) any interventional or surgical procedure of intracranial or extracranial arteries (e.g., angioplasty or carotid endarterectomy) within one month before the index stroke, or before any stroke recurrence during 1-year follow-up. Patients who received interventional or surgical procedure after stroke recurrence during follow-up were not excluded.

Demographics, NIH Stroke Scale (NIHSS), cardiovascular risk factors, blood pressure, laboratory test results were collected at baseline. Patients in the SOpHIA study [6] also had CTA exams at baseline where the percentage of luminal stenosis was measured using the Warfarin-Aspirin Symptomatic Intracranial Disease (WASID) method [7], and dichotomized as moderate (50%−69%) and severe (70%−99%).

### Stroke Mechanism Classification Based on Infarct Topography in DWI

**Fig. 1:**
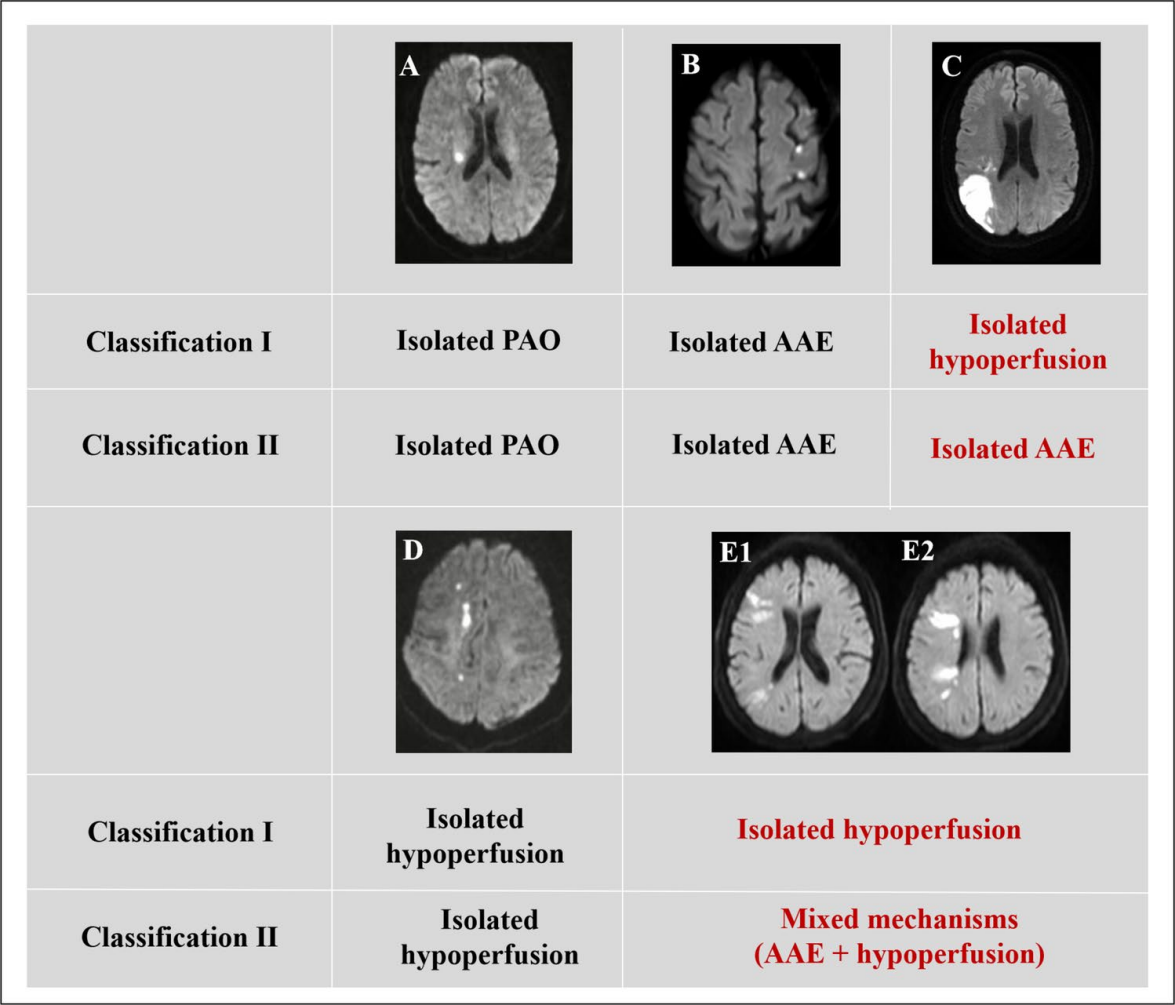
Two stroke mechanism classification systems based on infarct topography. Stroke mechanisms are classified as isolated parent artery atherosclerosis occluding penetrating artery (PAO), isolated artery-to-artery embolism (AAE), isolated hypoperfusion and mixed mechanisms, in both Classifications I and II. The difference between the two classification systems is the classification of cortical borderzone infarcts respectively as hypoperfusion and AAE in the two systems, hence the differences in the classifications in cases C&E. **A**: An isolated subcortical infarct in the penetrating artery territory, classified as isolated PAO in both classification systems. **B**: Small, multiple cortical infarcts, classified as isolated AAE in both classifications. **C**: Wedge-shaped infarct in the posterior cortical borderzone, respectively classified as isolated hypoperfusion and isolated AAE in Classification I and Classification II. **D**: Small chain-like infarcts in the internal borderzone, classified as isolated hypoperfusion in both classification systems. E1&E2: Wedge-shaped infarcts in the anterior and posterior cortical borderzone (E1&E2) and small infarcts in the internal borderzone (E2), respectively classified as isolated hypoperfusion in Classification I and mixed mechanisms of AAE + hypoperfusion in Classification II

Probable anterior-circulation stroke mechanisms at baseline were classified with two classification systems (Classifications I and II), according to the infarct topography (location, shape, size and number) in DWI (Fig. 1). Data on the infarct topography and Classification I were adopted from our previous study, with an excellent intra-rater reproducibility (kappa = 0.853; 95% confidential interval [CI], 0.785–0.920) and substantial inter-rater reproducibility (kappa = 0.791; 95% CI, 0.713–0.869) [2]. Classification II was a modified version of Classification I, also based on the infarct topography data from our previous study. The only difference between Classifications I and II was that CBZ infarcts were considered to be caused by hypoperfusion in Classification I but AAE in Classification II.

### Classification I

1. ***Isolated PAO:*** Isolated subcortical infarct (usually with a diameter ≤ 20 mm) within the territory of penetrating artery, extending from the lower portion of the basal ganglia, with a ≥ 50% atherosclerotic stenosis in the adjacent parent artery [8].
2. ***Isolated AAE***: Small multiple cortical infarcts or wedge-shaped territorial infarct(s), not involving CBZ regions (the cortical and adjacent subcortical regions between MCA and anterior cerebral artery territories, or MCA and posterior cerebral artery territories).
3. ***Isolated hypoperfusion***: IBZ infarcts, which were small, scattered (≥ 3 lesions) chain-like infarcts or large, confluent cigar-like infarct, along or slightly above the lateral ventricle (the junctional regions between superficial and perforating arteries of MCA), and/or CBZ infarct(s).
4. ***Mixed mechanisms***: Coexisting 2 or more mechanisms.

### Classification II

1. ***Isolated PAO:*** Same with Classification I.
2. ***Isolated AAE***: In addition to small multiple cortical infarcts or wedge-shaped territorial infarct(s), CBZ infarcts were also classified as AAE.
3. ***Isolated hypoperfusion:*** Only IBZ infarcts were classified as hypoperfusion, while CBZ infarcts were considered to be caused by AAE as mentioned above.
4. ***Mixed mechanisms***: Coexisting 2 or more mechanisms.

In this study, the stroke mechanisms were analyzed by the 4 categories, which were also analyzed as a binary variable by the presence of hypoperfusion as a stroke mechanism, since the modification may significantly affect this category that has been associated with higher stroke risks [2].

### Treatment and Follow-Up

All patients received medical treatment according to the guidelines [9], including antiplatelet, statin therapy and vascular risk factor management. Patients at Prince Wales Hospital were followed up at 1, 3, 6, 9 and 12 months by neurologists at an out-patient clinic. Patients at the other hospital were followed up at 1 year at neurology outpatient clinics or by telephone. At both centers, any recurrent ischemic stroke in the same territory (SIT) of the index artery within 90 days and 1 year, and the date of recurrence, were recorded, defined as newly developed neurological deficits accompanied by any new infarct(s) revealed on CT or MRI; or diagnosed by a neurologist with newly developed neurological deficits lasting more than 24 h, if there was no CT/MRI at recurrence [6].

### Statistical Analysis

Statistical analyses were conducted using IBM SPSS Statistics 25.0. Two-sided p < 0.05 was defined as statistically significant. Continuous variables and categorical variables were respectively presented as medians (interquartile ranges, IQR) and numbers (percentage). McNemar’s tests were conducted to compare the proportion of stroke mechanisms at baseline in the two classification systems. Mann–Whitney U tests, Chi-square tests or Fisher’s exact tests were conducted to compare baseline characteristics and medications at discharge between patients with and without recurrent SIT within 90 days and 1 year.

Survival analyses were conducted for recurrent SIT within 90 days and 1 year. Kaplan–Meier curves were plotted to present the cumulative probabilities of recurrent SIT within 90 days or 1 year, and the differences in stroke risks by stroke mechanisms were examined by log-rank tests. Receiver operating characteristics (ROC) curves were plotted and areas under the curve (AUC) were calculated for the predictive values of the two classification systems for each outcome, which were compared by Z test (DeLong method). In these analyses, the stroke mechanisms were analyzed first as a 4-category variable, and then as a binary variable by the presence of hypoperfusion.

### Data Availability

Data related to the current study are available from the corresponding author on reasonable request.

## Results

### Patients’ Characteristics

**Fig. 2:**
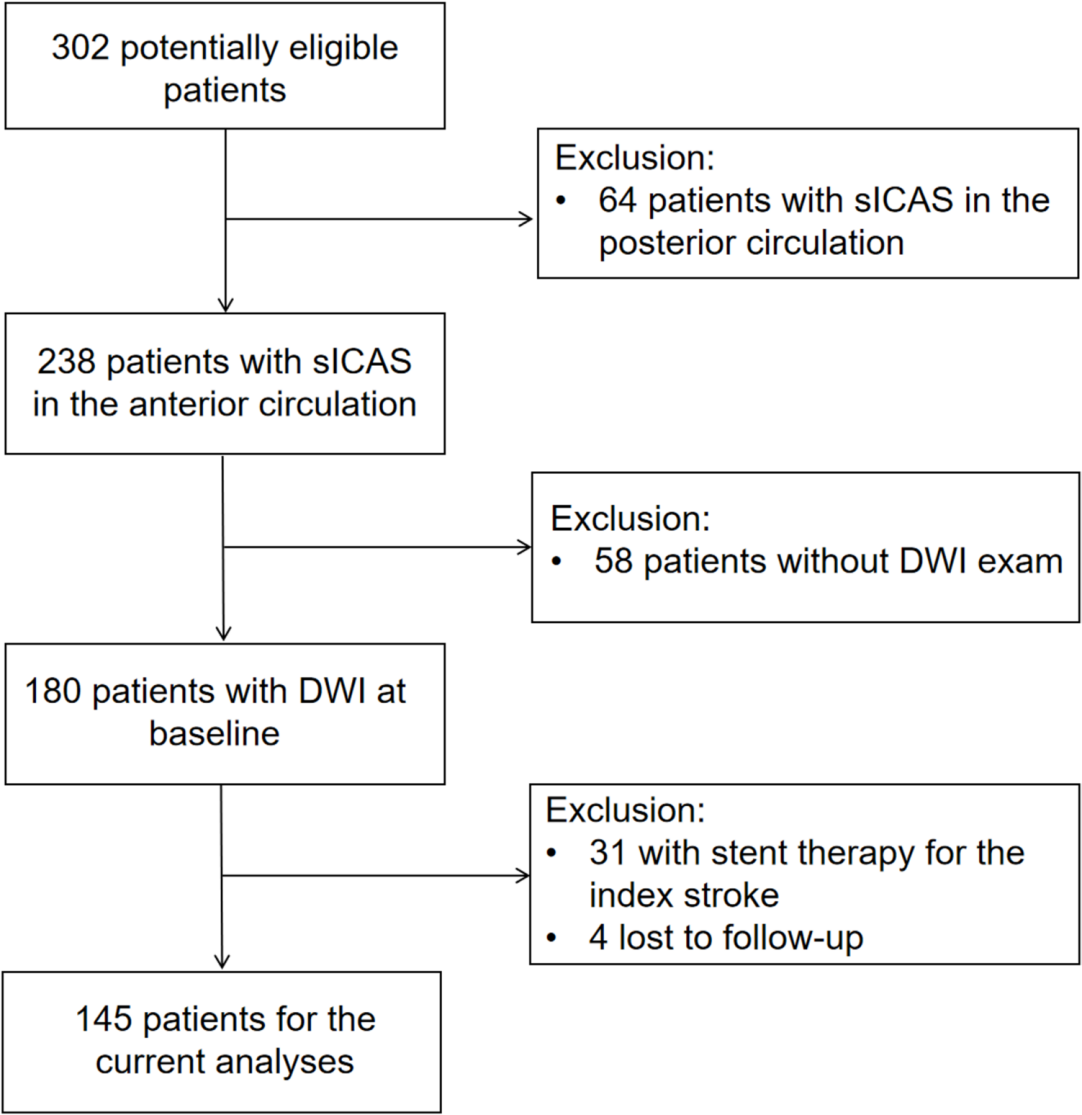
Flow chart of patient screening for this study. sICAS, symptomatic intracranial atherosclerotic stenosis; DWI, diffusion-weighted imaging

Among 302 potential eligible patients for SOpHIA, 64 patients with posterior-circulation sICAS, 58 without baseline DWI, 31 with stent therapy for the index stroke and 4 lost to follow-up were excluded (flow chart in Fig. 2). Finally, 145 medically treated patients with anterior-circulation sICAS were included in the current analyses, with a median age of 61 (IQR 53–69) years and 101 (69.7%) being males (Table 1).

**Table 1:** Comparisons of patients’ characteristics by 90-day and 1-year recurrent SIT

	Overall (n = 145)	90 days			1 year		
		Recurrent SIT ( +) (n = 11)	Recurrent SIT (–) (n = 134)	P value	Recurrent SIT ( +) (n = 19)	Recurrent SIT (–) (n = 126)	P value
Baseline demographics							
Age, years	61 (53–69)	57 (49–69)	62 (53–69)	0.455	57 (49–72)	62 (53–69)	0.523
Male	101 (69.7)	4 (36.4)	97 (72.4)	0.019	9 (47.4)	92 (73.0)	0.023
Smoking	72 (49.7)	2 (18.2)	70 (52.2)	0.030	6 (31.6)	66 (52.4)	0.091
Hypertension	89 (61.4)	9 (72.7)	81 (60.4)	0.530	15 (78.9)	74 (58.7)	0.092
Diabetes mellitus	51 (35.2)	3 (27.3)	48 (35.8)	0.747	7 (36.8)	44 (34.9)	0.870
Hyperlipidemia	79 (54.5)	7 (63.6)	72 (53.7)	0.526	11 (57.9)	68 (54.0)	0.749
Prior ischemic heart disease	5 (3.4)	0 (0.0)	5 (3.8)	1.000	1 (5.3)	4 (3.2)	0.515
Prior stroke/TIA^*^	17 (11.7)	4 (36.4)	13 (9.8)	0.027	5 (26.3)	12 (9.6)	0.051
NIHSS at admission^†^	3 (1–5)	3 (0–3)	3 (1–5)	0.427	3 (1–4)	3 (1–5)	0.986
SBP at admission, mmHg	150 (135–167)	150 (136–160)	150 (135–168)	0.593	150 (136–163)	150 (134–168)	0.541
DBP at admission, mmHg	84 (76–93)	80 (70–92)	84 (78–94)	0.358	80 (70–92)	84 (78–94)	0.310
Lab test results at baseline							
Fasting glucose, mmol/L	5.6 (5.0–7.5)	6.0 (5.3–7.6))	5.6 (4.9–7.4)	0.456	6.0 (5.2–7.6)	5.6 (4.9–7.4)	0.557
HbA1c, %	6.2 (5.6–7.3)	6.4 (5.6–6.8)	6.1 (5.6–7.4)	0.970	6.5 (5.6–7.3)	6.1 (5.7–7.3)	0.612
Triglycerides, mmol/L	1.4 (1.0–2.0)	1.2 (1.1–1.7)	1.5 (1.0–2.1)	0.367	1.2 (1.0–1.3)	1.5 (1.0–2.0)	0.513
HDL-C, mmol/L	1.1 (1.0–1.3)	1.2 (0.7–1.5)	1.1 (1.0–1.3)	0.943	1.1 (0.8–1.3)	1.1 (1.0–1.3)	0.327
LDL-C, mmol/L	3.1 (2.3–3.9)	2.7 (1.9–3.7)	3.1 (2.3–4.0)	0.461	2.6 (1.9–3.6)	3.3 (2.3–4.0)	0.078
Luminal stenosis of sICAS							
Degree of stenosis, %	70 (57–80)	76 (64–99)	69 (56–77)	0.047	69 (56–77)	69 (55–79)	0.161
Severe (70%−99%) stenosis	74 (51)	8 (72.7)	66 (49.3)	0.134	12 (63.2)	62 (49.2)	0.257
Medications at discharge							
Antiplatelet	141 (97.2)	11 (100.0)	130 (97.0)	1.000	19 (100.0)	122 (96.8)	1.000
Mono antiplatelet	90 (63.8)	5 (45.5)	85 (65.4)	0.205^‡^	10 (52.6)	80 (65.6)	0.275^‡^
Dual antiplatelets	51 (31.2)	6 (54.4)	45 (34.6)		9 (34.4)	9 (47.4)	
Statin	132 (91.0)	10 (90.9)	122 (91.0)	1.000	18 (94.7)	114 (90.5)	1.000
Antihypertensive	68 (46.9)	5 (45.5)	63 (47.0)	0.921	12 (63.2)	56 (44.4)	0.128
Antidiabetics	42 (29.0)	3 (27.3)	39 (29.1)	1.000	6 (31.6)	36 (28.6)	0.788

^*^ Missing in 2 patients

^†^ Missing in 1 patient

^‡^ Mono antiplatelet versus dual antiplatelet

**Abbreviations:** *SIT* ischemic stroke in the same territory, *TIA* transient ischemic attack, *NIHSS* National Institutes of Health Stroke Scale, *SBP* systolic blood pressure, *DBP* diastolic blood pressure, *HbA1c* glycosylated hemoglobin, *HDL-C* high-density lipoprotein cholesterol, *LDL-C* low-density lipoprotein cholesterol

The numbers of patients with isolated PAO, isolated AAE, isolated hypoperfusion, and mixed mechanisms were respectively 28 (19.3%), 18 (12.4%), 52 (35.9%), and 47 (32.4%) in Classification I, which were respectively 28 (19.3%), 43 (29.7%), 30 (20.8%), and 44 (30.3%) in Classification II. There were significant differences in the proportions of isolated AAE and isolated hypoperfusion (p < 0.001). When classified by the presence of hypoperfusion as a stroke mechanism (with or without other mechanisms), 99 (68.3%) patients had hypoperfusion in Classification I and 74 (51.0%) in Classification II, which was significantly different between the two systems (p < 0.001). More details are presented in Tables 2 and 3 and Supplemental Figure S1.

**Table 2:** Stroke mechanisms and stroke risks in 2 classification systems

	Overall (n = 145)	90 days			1 year		
		Recurrent SIT ( +) (n = 11)	Recurrent SIT (–) (n = 134)	Log-rank p value	Recurrent SIT ( +) (n = 19)	Recurrent SIT (–) (n = 126)	Log-rank p value
Classification I				0.120			0.024
Isolated PAO	28 (19.3)	0 (0.0)	28 (19.3)		0 (0.0)	28 (19.3)	
Isolated AAE	18 (12.4)	0 (0.0)	18 (12.4)		1 (5.6)	17 (94.4)	
Isolated hypoperfusion	52 (35.9)	5 (9.6)	47 (90.4)		7 (13.5)	45 (86.5)	
Mixed mechanisms	47 (32.4)	6 (12.8)	41 (87.2)		11 (23.4)	36 (76.5)	
Classification II				0.009			0.009
Isolated PAO	28 (19.3)	0 (0.0)	28 (19.3)		0 (0.0)	28 (19.3)	
Isolated AAE	43 (29.7)	0 (0.0)	43 (29.7)		3 (7.0)	40 (93.0)	
Isolated hypoperfusion	30 (20.8)	4 (13.3)	26 (86.7)		5 (16.7)	25 (83.3)	
Mixed mechanisms	44 (30.3)	7 (15.9)	37 (84.1)		11 (25.0)	33 (75.0)	

**Abbreviations:** *PAO* parent artery atherosclerosis occluding penetrating artery, *AAE* artery-to-artery embolism, *SIT* ischemic stroke in the same territory

**Table 3:** Presence of hypoperfusion as a baseline stroke mechanism and stroke risks in 2 classification systems

	Overall (n = 145)	90 days			1 year		
		Recurrent SIT ( +) (n = 11)	Recurrent SIT (–) (n = 134)	Log-rank p value	Recurrent SIT ( +) (n = 19)	Recurrent SIT (–) (n = 126)	Log-rank p value
Classification I				0.020			0.009
Hypoperfusion ( +)	99 (68.3)	11 (11.1)	88 (88.9)		18 (18.2)	81 (81.8)	
Hypoperfusion (–)	46 (31.7)	0 (0.0)	46 (100)		1 (2.2)	45 (97.8)	
Classification II				0.001			0.002
Hypoperfusion ( +)	74 (51.0)	11 (14.8)	63 (85.1)		16 (21.6)	58 (76.3)	
Hypoperfusion (–)	71 (49.0)	0 (0.0)	71 (100)		3 (4.2)	40 (93.0)	

**Abbreviations:** *SIT *ischemic stroke in the same territory

Eleven (7.6%) patients had recurrent SIT within 90 days, who were less likely to be males (p = 0.019) and current smokers (p = 0.030) but more likely to have prior ischemic stroke/transient ischemic attack (TIA, p = 0.027) and more severe luminal stenosis (p = 0.047) than those without SIT (Table 1). Nineteen (13.1%) patients had recurrent SIT within 1 year, who were less likely to be males (p = 0.023) than those without SIT (Table 1). Other baseline characteristics and medications prescribed at discharge were comparable between those with and without SIT.

### Risks of SIT by 4 Categories of Baseline Stroke Mechanisms in Classifications I and II

**Fig. 3:**
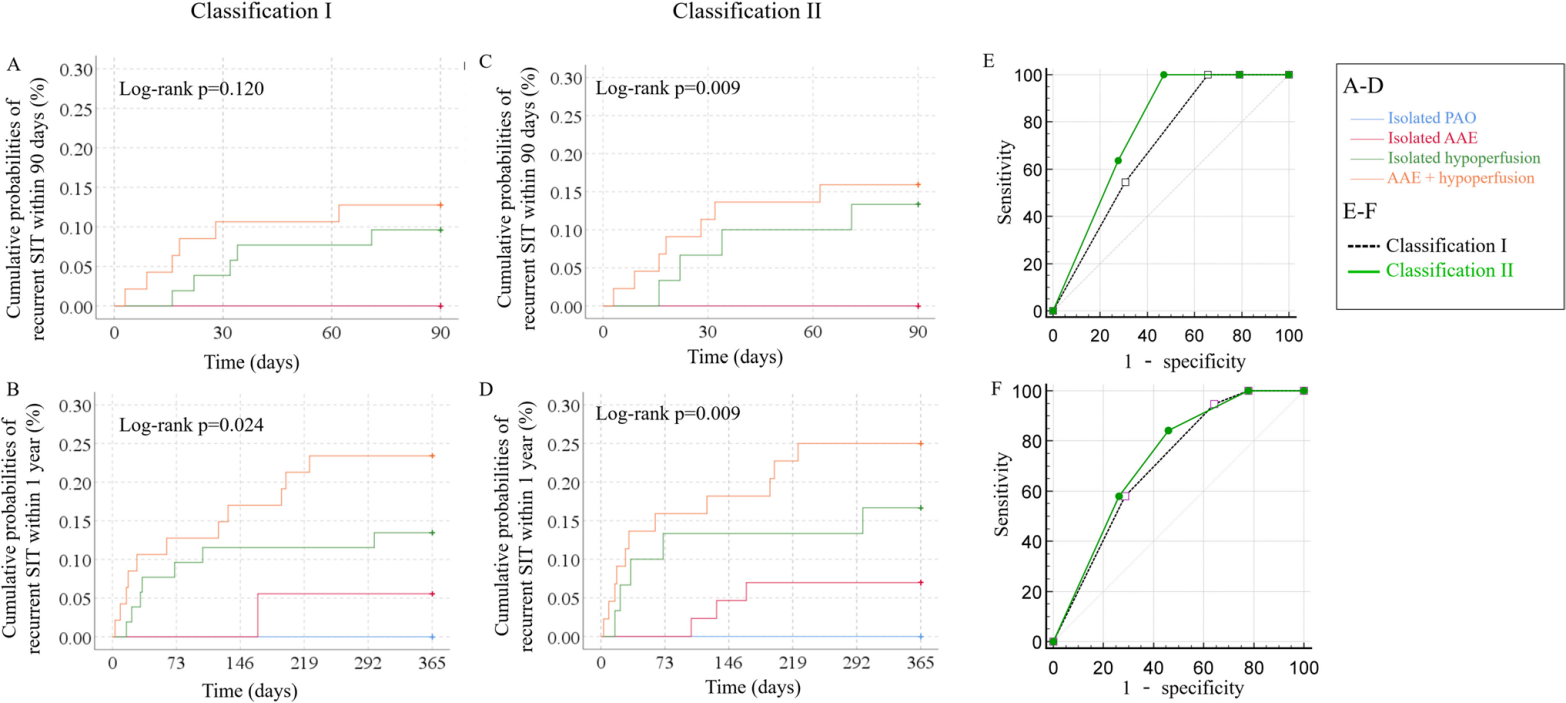
Cumulative probabilities of recurrent ischemic stroke in the same territory (SIT) within 90 days and 1 year by different baseline stroke mechanisms. A&B: In Classification I, cumulative probability of recurrent SIT was higher in patients with artery-to-artery embolism (AAE) + hypoperfusion than those with isolated hypoperfusion or other mechanisms (log-rank p = 0.024) within 1 year, but not in the first 90 days (log-rank p = 0.120). **C**&**D**: In Classification II, cumulative risks of recurrent SITs were significantly different by the 4 categories of baseline stroke mechanisms in both 90 days (log-rank p = 0.009) and 1 year (log-rank p = 0.009). **E**&**F**: The area under the curve of Classification II was significantly larger than that of Classification I (0.776 vs 0.698, Z = 2.18, p = 0.029), in predicting the risk of 90-day SIT, while the two classifications had comparable AUCs in predicting the 1-year SIT risk (0.731 vs 0.709, Z = 0.57, p = 0.572). PAO, parent artery atherosclerosis occluding penetrating artery

In Classification I, among the 11 (7.6%) patients with recurrent SIT within 90 days, 6 (4.1%) had AAE + hypoperfusion and 5 (3.4%) had isolated hypoperfusion at baseline. Among the 19 (13.1%) patients with recurrent SIT within 1 year, 11 (7.6%), 7 (4.8%), and 1 (0.7%) respectively had AAE + hypoperfusion, isolated hypoperfusion, and isolated AAE at baseline. Risks of recurrent SIT within 90 days were not significantly different among the 4 categories (12.8% in AAE + hypoperfusion, 9.6% in isolated hypoperfusion, 0% in isolated AAE and isolated PAO; log-rank p = 0.120; Table 2 and Fig. 3A). However, the 1-year risks of recurrent SIT were significantly higher in patients with AAE + hypoperfusion than those with other mechanisms (23.4% in AAE + hypoperfusion, 13.5% in isolated hypoperfusion, 5.6% in isolated AAE, and 0% in isolated PAO; log-rank p = 0.024; Table 2 and Fig. 3B).

In Classification II, among 11 (7.6%) patients with recurrent SIT within 90 days, 7 (4.8%) had AAE + hypoperfusion and 4 had (2.8%) isolated hypoperfusion at baseline. By 1 year, 11 (7.6%), 5 (3.4%), and 3 (2.1%) patients with AAE + hypoperfusion, isolated hypoperfusion or isolated AAE at baseline had recurrent SITs. The risks of recurrent SITs were significantly different by the 4 categories of baseline stroke mechanisms in both 90 days (15.9% in AAE + hypoperfusion, 13.3% in isolated hypoperfusion; 0.0% in isolated AAE; 0.0% in isolated PAO; log-rank p = 0.009; Table 2 and Fig. 3C) and 1 year (25.0% in AAE + hypoperfusion; 16.7% in isolated hypoperfusion,; 7.0% in isolated AAE; 0.0% in isolated PAO; log-rank p = 0.009; Table 2 and Fig. 3D).

The AUC of Classification II with 4 categories of stroke mechanisms was significantly larger than that of Classification I (0.776 vs 0.698, Z = 2.18, p = 0.029; Fig. 3E), in predicting the risk of 90-day SIT, while the two classifications had comparable AUCs in predicting the 1-year SIT risk (0.731 vs 0.709, Z = 0.57, p = 0.572; Fig. 3F).

### Risks of SIT by Presence of Hypoperfusion as a Baseline Stroke Mechanism in Classifications I and II

**Fig. 4:**
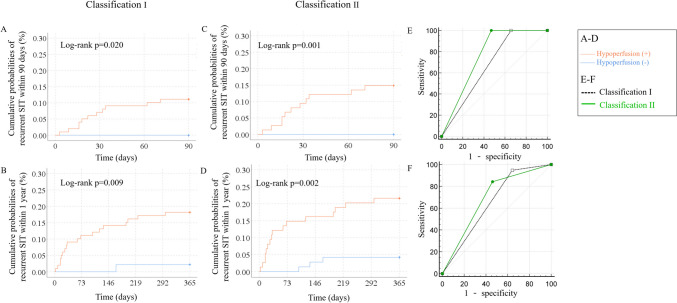
Cumulative probabilities of recurrent ischemic stroke in the same territory (SIT) within 90 days and 1 year by the presence of hypoperfusion as a baseline stroke mechanism. **A**&**B**: In Classification I, patients with hypoperfusion as a baseline stroke mechanism had a significantly higher stroke risk of recurrent SIT in 90 days (11.1% versus 0.0%; log-rank p = 0.020) and 1 year (18.2% versus 2.2%; log-rank p = 0.009). **C**&**D**: In Classification II, patients with hypoperfusion had a significantly higher stroke risk in both 90 days (14.8% versus 0.0%; log-rank p = 0.001) and 1 year (21.6% versus 4.2%; log-rank p = 0.002). **E**&**F**: The area under curve of Classification II by presence of hypoperfusion was significantly larger than Classification I in predicting the risk of 90-day SIT (0.765 vs 0.672, Z = 5.52, p < 0.001), which were similar for 1-year SIT (0.691 vs 0.652, Z = 0.96, p = 0.335)

All of the 11 patients with recurrent SIT within 90 days had hypoperfusion as a baseline stroke mechanism by Classification I. Among the 19 patients with 1-year recurrent SITs, 18 had hypoperfusion as a baseline stroke mechanism by Classification I. Patients with hypoperfusion at baseline had a higher risk of recurrent SIT in 90 days (11.1% versus 0.0%; log-rank p = 0.020; Table 3 and Fig. 4A) and 1 year (18.2% versus 2.2%; log-rank p = 0.009; Table 3 and Fig. 4B) than those without.

All of the 11 patients with recurrent SIT within 90 days had hypoperfusion as a baseline stroke mechanism by Classification II. Among the 19 patients with 1-year recurrent SITs, 16 had hypoperfusion as a baseline stroke mechanism by Classification II. The risks of recurrent SITs were higher in patients with hypoperfusion than those without, in both 90 days (14.8% versus 0.0%; log-rank p = 0.001; Table 3 and Fig. 4C) and 1 year (21.6% versus 4.2%; log-rank p = 0.002; Table 3 and Fig. 4D).

The AUC of Classification II by presence of hypoperfusion was significantly larger than that of Classification I (0.765 vs 0.672, Z = 5.52, p < 0.001; Fig. 4E) in predicting the risk of 90-day SIT, which were comparable in predicting the risk of 1-year recurrent SIT (0.691 vs 0.652, Z = 0.96, p = 0.335; Fig. 4F).

## Discussion

While hypoperfusion was usually considered as the stroke mechanism for both IBZ and CBZ infarcts in anterior-circulation sICAS (e.g., in a previous stroke classification mechanism system, Classification I), we proposed a modified stroke mechanism classification system (Classification II) in this study, which considered AAE as the stroke mechanism for CBZ infarcts and hypoperfusion for IBZ infarcts. The proportions of AAE and hypoperfusion as stroke mechanisms were significantly different between the two classification systems. Irrespective of the stroke mechanism classification systems, we found that sICAS patients with hypoperfusion, especially those with hypoperfusion combined with AAE, had higher stroke risks than those with other stroke mechanisms, in the first 3 months and 1 year under modern medical treatment. The two classification systems had comparable predictive values for recurrent SIT within 1 year in sICAS patients, but Classification II had significantly higher predictive value for recurrent SIT within 90 days.

Intracranial atherosclerosis is prevalent worldwide, especially in Asian populations [10], with a considerable stroke risk despite contemporarily optimal medical treatment [4, 11]. Currently, sICAS patients are mostly treated under a uniform regimen, including antiplatelet, statin therapy and vascular risk factor management, while those with severe luminal stenosis (70–99%) or with minor stroke or high-risk TIA are considered as “high-risk” patients and given short-term dual antiplatelets [12]. However, according to previous studies, sICAS patients with different stroke mechanisms may have different risks of recurrent stroke under such medical treatment [2]. Therefore, recognition of the stroke mechanisms could be very important to identify “high-risk” sICAS patients. The acute infarct topology, which can be easily assessed in DWI that is commonly included in the MRI scanning protocol for ischemic stroke patients, has been used to determine probable stroke mechanisms in sICAS patients [2, 13, 14].

In patients with anterior-circulation sICAS, IBZ and CBZ infarcts have mostly been considered to be caused by hypoperfusion in previous studies and in clinical practice [2, 5]. Yet, there has been evidence suggesting a role of AAE in leading to CBZ infarcts in sICAS patients. In our previous study, over 50% of sICAS patients with CBZ infarcts had concomitant small cortical infarcts, suggesting possibly underlying AAE [4]. Moreover, our previous study did not find evidence of significant hemodynamic compromise in sICAS patients with isolated CBZ infarcts, using a CTA-based CFD model to simulate and gauge the hemodynamics [4]. Therefore, in this study, we proposed to reclassify CBZ infarcts from the “hypoperfusion” category to the “AAE” category in a modified stroke mechanism classification system, in the hope of more accurately stratifying the stroke risks in medically treated sICAS patients by the stroke mechanisms. The modified system (Classification II) did exhibit higher predictive values for short-term (3 months) recurrent SITs than the previous version (Classification I), while the two systems had comparable predictive values for long-term (1 year) recurrent SITs. Since most recurrent strokes in sICAS patients occur in the first few weeks or months after the index stroke [15, 16], Classification II may yield higher values in identifying high-risk patients and informing early interventions.

The different predictive values of the two classification systems for 3-month SITs may be explained by the different trajectories of stroke recurrence in sICAS patients with true “hypoperfusion” and “AAE”, under current medical treatment. Those with true “hypoperfusion” at baseline might be more prone to recurrent SIT early after an index stroke, if there was no interventional or medical treatment to restore or improve the cerebral perfusion [4]. Particularly, blood pressure control, usually initiated within a few days after an index stroke, may further impair the cerebral perfusion in such patients and increase the stroke risk in the first few months [2]. On the other hand, short-term (up to 90 days) dual antiplatelets in some patients and high-intensity statin therapy in all patients without contraindications may effectively prevent SITs in sICAS patients with true “AAE” as a stroke mechanism, particularly within the first few month when medication compliance is usually better than later [17]. In this study, the modified Classification II reclassified CBZ infarcts from “hypoperfusion” to “AAE”, which may have more accurately differentiated true “hypoperfusion” and “AAE” patients with different risks of SIT within the first few months (as explained above), hence the higher predictive values of Classification II than I for the 90-day SIT risks. This stood when analyzing the stroke mechanisms as 4 categories, as well as by the presence of “hypoperfusion”. The two classification systems had comparable predictive values for the 1-year SIT risks in this study, since CBZ and IBZ infarcts bear similar risks of SITs beyond the first few months after an index stroke [4]. However, it should be noted that regardless of the classification methods, identifying the stroke mechanisms in sICAS is crucial, as different mechanisms are associated with different stroke risks.

In addition to providing a probably more reasonable stroke mechanism classification system for more accurate stroke risk stratification in sICAS patients, this study reinforced the need for testing more individualized treatments for sICAS patients with different stroke mechanisms, rather than using the current one-size-fits-all paradigm. For instance, long-term, stringent blood pressure control as recommended in current guidelines[18] may further impair the cerebral perfusion and increase the stroke risk in sICAS patients with hypoperfusion as a stroke mechanism [11], while it may worth testing the efficacy of endovascular treatment (that can restore or improve cerebral perfusion) for secondary stroke prevention in such patients [19]. On the other hand, it may worth testing longer-term dual antiplatelet and more intensive lipid-lowering therapies in those with AAE as a stroke mechanism, both of which therapies may help stabilize the sICAS lesion and reduce the risk of plaque rupture and AAE [17]. Moreover, hypoperfusion may reduce the capacity of clearing emboli and hence increase the risk of AAE [20, 21], which commonly exist as mixed stroke mechanisms in sICAS patients and associate with higher stroke risks under current medical treatment. Further studies are needed to explore for more effective secondary stroke prevention strategies in such patients.

There were some limitations. First, the sample size is relatively small, with small numbers of patients with the outcome events, which prevented multivariate analyses adjusting for other potential confounders. Particularly, some demographics, vascular risk factors and degree of luminal stenosis of the sICAS lesion may be associated with the 90-day SIT risk. Future larger-scale studies with adequate numbers of outcome events are warranted to verify the current findings. Second, in the current study, no patient with isolated PAO as a baseline stroke mechanism had recurrent SIT within 90 days or 1 year. This supported the effectiveness of current medical treatment for secondary stroke prevention in such patients, which, however, prevented the calculation of hazard ratios in survival analysis. In addition, in this study the stroke mechanism of isolated PAO was not equivalent to lacunar syndrome, while it was classified based on the infarct pattern without considering the clinical manifestations. It should be noted that although lacunar syndromes are highly suggestive of small cerebral infarctions (i.e., lacunar infarcts), it is not uncommon that patients could also present with lacunar syndromes with non-lacunar infarcts.[22] Third, although patients were all treated by the guidelines, we did not have data on medication compliance during follow-up, which may confound the findings. Fourth, the current study only investigated stroke mechanisms in patients with sICAS in the anterior circulation. By far, there has been no established or widely accepted criteria for classifying stroke mechanisms for posterior-circulation sICAS, which needs to be investigated in future studies. In addition, this study was conducted in Chinese patients only. With ethnic difference in ICAS prevalence between Asians and Caucasians [10], it is unknown whether the proportions of different stroke mechanisms are also different across populations. Therefore, the current findings need to be verified in other populations.

In conclusion, when classifying the stroke mechanisms in patients with anterior-circulation sICAS, considering AAE rather than hypoperfusion as the stroke mechanism for CBZ infarcts had significantly higher predictive value for early recurrent SITs under current medical treatment regimen. Therefore, this study provided a probably more reasonable stroke mechanism classification system (Classification II) for more accurate stroke risk stratification in sICAS patients than previous classification systems. With further validation of this classification system, studies are warranted to test for more effective secondary stroke prevention strategies for sICAS patients by the stroke mechanism.

## Supplementary Information

Below is the link to the electronic supplementary material.

Supplementary file1 (PDF 218 KB)

## Data Availability

No datasets were generated or analysed during the current study.
