# Supplementary material for: Stroke Mechanisms in Intracranial Atherosclerotic Disease: A Modified Classification System and Clinical Implications
Source: Transl Stroke Res. 2025 Mar 6;16(5):1655–65. doi: 10.1007/s12975-025-01338-0 (PMC12391173; doi:10.1007/s12975-025-01338-0)
Supplement: Supplementary file 1 — Supplementary file1 (PDF 218 KB) [file 12975_2025_1338_MOESM1_ESM.pdf]

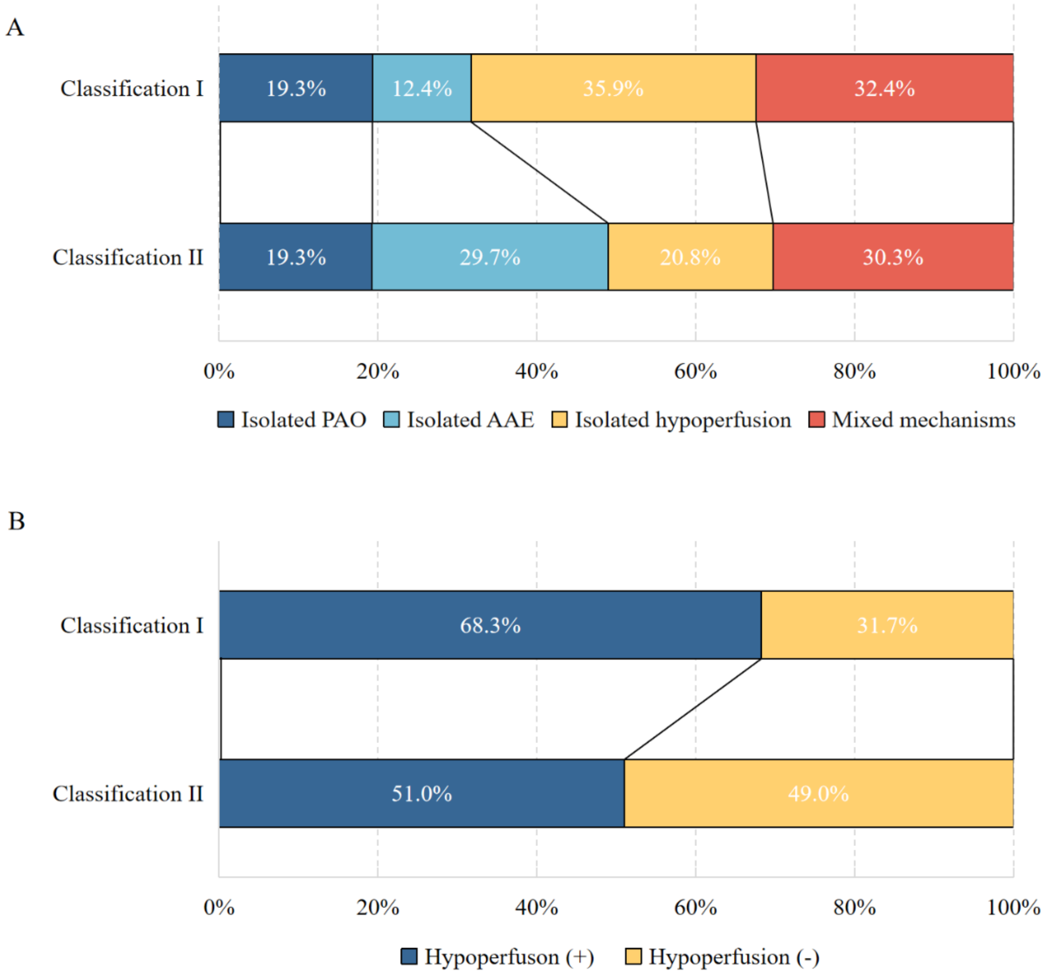

Supplemental Figure S1. Distribution of baseline stroke mechanisms in the two classification systems

A: Distribution of the 4 categories of stroke mechanisms in Classification I versus Classification II. There were significant differences in the proportions of isolated AAE and isolated hypoperfusion ($p<0.001$).

B: Significant difference in the proportions of hypoperfusion and other stroke mechanisms in Classification I versus Classification II ($p<0.001$).

PAO, parent artery atherosclerosis occluding penetrating artery; AAE, artery-to-artery embolism.
